# Analysis and Evaluation of Nutritional Intake and Nutrition Quotient of Korean Athletes with Disabilities in the Tokyo Paralympic Games

**DOI:** 10.3390/nu13103631

**Published:** 2021-10-16

**Authors:** Bogja Jeoung, Jiyoun Kim

**Affiliations:** Department of Exercise Rehabilitation & Welfare, Gachon University, Incheon 21936, Korea; bogja05@gachon.ac.kr

**Keywords:** nutrition, sports, national disabled athlete, nutritional status, nutrition quotient for adults

## Abstract

This study analyzed and evaluated the nutritional intake and nutrition quotient for adults (NQ-A) among 21 disabled national athletes preparing for the Tokyo Paralympic competition. A 24-h recall nutrition survey was conducted on the second day of training and one day of the weekend (holidays) to analyze daily nutritional intake. Dietary information was analyzed using the NQ-A questionnaire, which comprises 21 items. The athletes were divided into three groups based on the NQ-A score (High, Middle, Low). A comparative analysis of dietary intake of disabled athletes with the recommended dietary intake amount (RDA) was performed. The intake of carbohydrates (166.9%), proteins (112.3%), vitamin E (112.0%), thiamine (124.6%), riboflavin (100.2%), vitamin B6 (110.6%), vitamin B12 (120.7%), sodium (216.6%), phosphorus (118.3%), iron (146.5%), iodine (143.2%), and selenium (114.2%) was higher than the Korean-recommended amount. In particular, as the results of comparing dietary intake between the three groups showed, the low NQ-A score group had significantly lower intake compared to the %RDA for vitamin E (67.1%), C (26.3%), and Potassium (42.8%). However, with most nutrients, nutritional intake deficiency was not shown to be a problem.

## 1. Introduction

The Tokyo 2020 Paralympic Games will be the first Olympic Games to be held since the coronavirus disease (COVID-19) pandemic. As the game is being held under the ongoing COVID-19 pandemic, a year after the postponement, it is very important to control the condition of the players for their performance. Athletes participating in the Paralympic Games are athletes with physical and visual impairments, and are classified according to physical disabilities, such as visual impairment, limb deficits, strength and range impairments, short stature, hypertonia, and ataxia [1,2]. As a result, there are many restrictions on sensory and motor functions compared to those of able-bodied athletes.

An individual’s dietary strategies can significantly influence their physical performance [3,4]. Therefore, it is necessary to apply the principles of sports nutrition to optimize the health and performance outcomes of athletes [5]. However, Korean Paralympic athletes are complaining that they are experiencing difficulties in receiving training support, such as a training schedule and diet management, contrary to the original plan, as their admission to the training center is delayed after the selection of the national team for each event due to COVID-19.

Nutrition management for athletes should be differentiated according to sports, exercise frequency, and intensity, and professional dietary planning and provision for sufficient nutrition to improve basic athletic performance is an important factor [6]. Adequate nutrition ensures energy generation and recovery [7], whereas insufficient nutrition can increase the risks of injury and disease and decrease training adaptation and performance [8]. Local and international studies on the importance of nutrition care for athletes’ performance have been published [9,10]. However, the management and research on athletes with disabilities remain insufficient.

South Korea’s parasports are aiming to rank 20th at the Tokyo Paralympics, with its world-class level of boccia, table tennis, shooting, and archery [11]. This study included the national team players who are currently preparing for the Tokyo Paralympics and have not received nutrition education or controlled nutrition care until now.

This is the first study in Korea that conducted a nutrition survey on national athletes with disabilities. This study aimed to analyze the nutritional balance of athletes with disabilities by examining their nutritional intake.

## 2. Materials and Methods

### 2.1. Study Design and Participants

This study aimed to record the dietary intake and nutrition index of athletes with disabilities who will participate in the Tokyo Paralympic Games and to evaluate their nutritional balance and dietary quality. This study included 26 national athletes with disabilities who were selected through the recommendation of the Korea Sports Council for the Disabled and agreed to participate in a survey on nutritional intake and nutrition index. The purpose and contents of this study were explained for 14 days from 18 September to 1 October 2020, and the survey was conducted after receiving written consent. Of 26 athletes, five were excluded due to incomplete data collection during the dietary intake survey.

Finally, 21 national athletes with disabilities and without missing data collection or data utilization were included. The general characteristics of the participating athletes are presented in Table 1. This study was approved by the Gachon University Bioethics Review Committee (IRB No: 1044396-201910-HR181-01).

### 2.2. Dietary Intake Survey

After fully explaining how the dietary intake survey will be conducted, the subjects were required to prepare their daily nutrient intake using the 24-h recall method.

The dietary survey recorded the intake during the second day of training and one day of the weekend (holidays). The name and amount of food were recorded, and the consumption of drinks and snacks was recorded without omission. In addition, to reduce errors in nutritional evaluation, photos of meals were recorded. Daily nutrient intake was analyzed using CANPro 5.0 (Korea Exercise Nutrition Society, Seoul, Korea), a nutrition evaluation program developed by the Korea Nutrition Society. CANPro 5.0 was created using the Food Ingredients Table of the Korean Nutrition Society as a reference. It contains 3986 food types and can be evaluated and analyzed with 48 basic nutrients, 34 fatty acids, and 21 amino acids. The professional version used in this study is a research tool used by nutrition-related professors, graduate students, food and drug research institutes, doctors in hospitals, public health centers, nutritionists, etc., to determine the nutritional status of individuals or groups. Dietary intake was compared and analyzed using the percentage of the Dietary Reference Intakes for Koreans (KDRIs, 2020; http://www.kns.or.kr/FileRoom/FileRoom_view.asp?idx=108&BoardID=Kdr; accessed on 8 April 2021) between the gender and age groups. Nutrient-specific nutrition criteria, recommended daily allowance (RDA), and energy without recommended intake were compared and analyzed based on adequate intake (AI). The percentage of the RDA value is expressed as percentage of RDA (%RDA).

The formula for calculating %RDA is as follows: %RDA = Nutrition intake/RDA(sex, age) × 100.

### 2.3. Nutrition Quotient for Adults (NQ-A)

The NQ-A, developed by the Korean Nutrition Society in 2018, evaluates the quality and nutritional status of meals using a simple checklist consisting of evaluation items on food intake and dietary behavior [9]. The NQ-A questionnaire is divided into four detailed factors: balance, diversity, moderation, and dietary behavior for 21 items. The first 12 items included are the following: consumption of vegetable side dishes, fruits, milk and dairy products, soy or soy products, eggs, fish, nuts, ramen, fast food, sweet or greasy bread, sweet drinks, and water intake frequency. The other nine items include breakfast, eating out or delivery, late-night snack, picky eating, 30 min before eating, nutrition indicating, nutrition. The higher the score in each area, the more positive it is, and the higher the frequency in the areas of balance, diversity, and dietary behavior, the higher the score. However, for moderation areas, the lower the frequency, the higher the score.

The NQ-A area-specific levels were determined and divided into three groups depending on the total score: 58.9–100 points, high-score group (75 to 100 percentile); 47.1–58.8 points, middle-score group (25 to <75 percentile); and 0–47.0 points, low-score group (0 to <25 percentile).

### 2.4. Statistical Analysis

The findings of this study were analyzed using IBM SPSS (version 21.0; IBM Corp., Armonk, NY, USA). Statistical data are presented as mean ± SE (standard error of the mean). Significant differences (at *p* < 0.05) between NA-Q group means were determined by one-way analysis of variance, followed by Scheffe’s post hoc test.

## 3. Results

### 3.1. Comparison of the Average Dietary Intake and %RDA of Korean Nationals with Disabilities

The classification of ratings for the number of comparisons between the average dietary intake and percentage of Korean-recommended quantities of subjects in this study are shown in Table 2.

**Figure 1:**
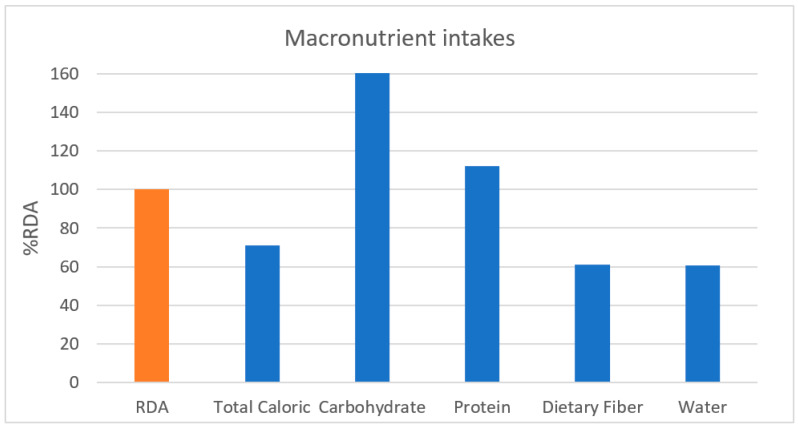
Macronutrient intakes. RDA, recommended daily allowance; %RDA, the percentage of the RDA value is expressed as percentage of RDA; %RDA = nutrition intake/RDA(sex, age) × 100.

A comparative analysis of macronutrient intakes showed (Figure 1) that the total intake of calories (70.9%), dietary fiber (61.1%), and water (60.6%) were smaller than the %RDA of Koreans, while carbohydrates (166.9%) and proteins (112.3%) were higher than the %RDA of Koreans.

**Figure 2:**
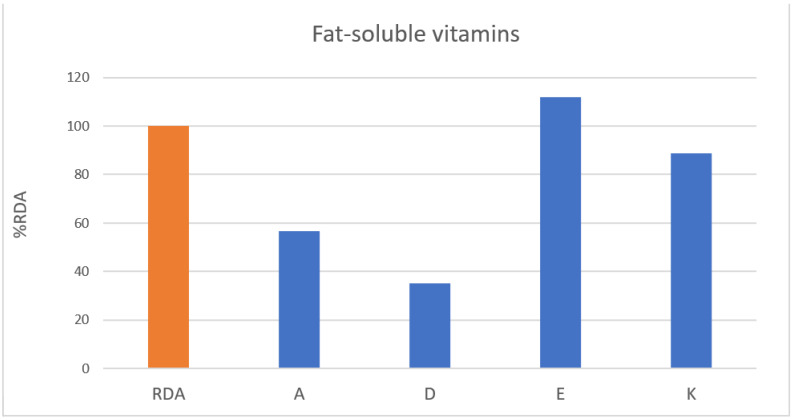
Fat-soluble vitamins. RDA, recommended daily allowance; %RDA, the percentage of the RDA value is expressed as percentage of RDA; %RDA = nutrition intake/RDA(sex, age) × 100.

**Figure 3:**
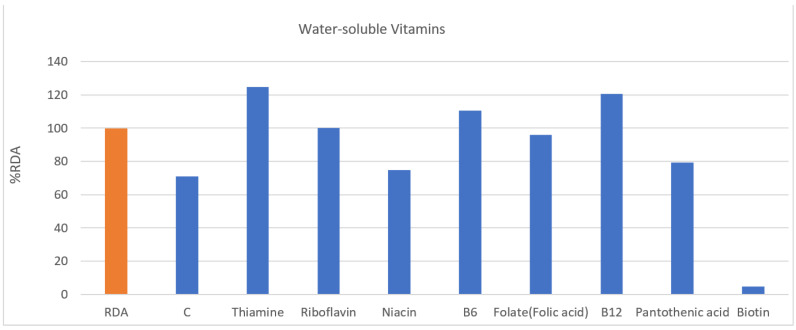
Water-soluble vitamins. RDA, recommended daily allowance; %RDA, the percentage of the RDA value is expressed as percentage of RDA; %RDA = nutrition intake/RDA(sex, age) × 100.

In fat-soluble vitamins (Figure 2), all but vitamin E (112.0%) was less than that in %RDA of Koreans. In water-soluble vitamins (Figure 3), they were taking sufficient amounts beyond the recommended amount of thymine (124.6%), riboflavin (100.2%), B6 (110.6%), and B12 (96.7%), while folate (folic acid) (96.0%) was taken close to the recommended amount. In addition, vitamin C (70.9%), niacin (74.8%), pantothenic acid (79.2%), and biotin (4.9%) showed lower intake than %RDA of Koreans.

**Figure 4:**
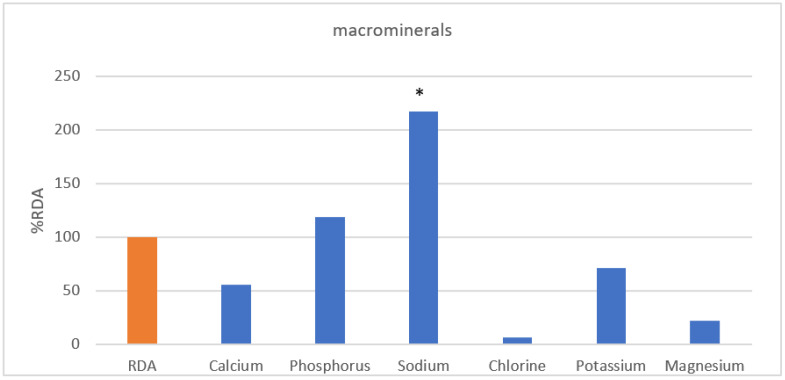
Macrominerals. * Indication of nutrients whose intake exceeds the upper limit. RDA, recommended daily allowance; %RDA, the percentage of the RDA value is expressed as percentage of RDA; %RDA = nutrition intake/RDA(sex, age) × 100.

**Figure 5:**
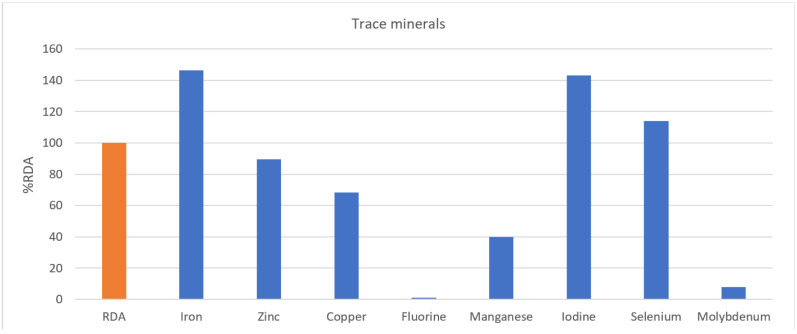
Trace minerals. RDA, recommended daily allowance; %RDA, the percentage of the RDA value is expressed as percentage of RDA; %RDA = nutrition intake/RDA(sex, age) × 100.

In Figure 4, sodium intake (216.6%) in large amounts of inorganic substances was more than twice as high as the standard, and phosphorus (118.3%) was higher than the %RDA of Koreans. In contrast, it was confirmed that calcium (55.74%), chlorine (6.3%), potassium (74.3%), and magnesium (22.0%) were less than the %RDA of Koreans. In trace minerals (Figure 5), iron (146.5%), iodine (143.2%), and selenium (114.2%) were higher than %RDA of Koreans, while zinc (89.6%), copper (68.3%), fluorine (1.2%), manganese (39.9%), and molybdenum (7.8%) were lower.

### 3.2. Comparison of Nutrition Quotient for Adults (NQ-A) and Scores by Detailed Factors of Korean Nationals with Disabilities

As the results of comparing the detailed elements between NQ-A score grades showed (Table 3), there was a significant difference between score groups in overall NQ-A (*p* < 0.001), diversity (*p* < 0.001), and dietary behavior (*p* < 0.001). In addition, as a result of the post hoc test showed, there was a significant difference between the higher score group (*p* < 0.001) and the intermediate score group (*p* < 0.003) in diversity compared to the lower score group. Furthermore, there was significant difference between the middle score group (*p* < 0.017) and the lower score group (*p* < 0.001) compared to the higher score group in dietary behavior.

### 3.3. Comparative Analysis of %RDA According to Nutrition Quotient for Adults (NQ-A) Score Groups of National Athletes with Disabilities

Table 4 shows the comparison of %RDA according to the NQ-A score groups in this study. The total calorie intake (*p* < 0.020), dietary fiber (*p* < 0.019), fat-soluble vitamin E (*p* < 0.015), water-soluble vitamin C (*p* < 0.010), thiamine (*p* < 0.012), phosphate (*p* < 0.010), potassium (*p* < 0.009), and magnesium (*p* < 0.005) were noted. As a result of the post hoc test, significant differences were observed between the high- and middle-score groups in total calorie intake and dietary fiber (*p* < 0.046, *p* < 0.046); between the high- and low-score groups in vitamin E (*p* < 0.020); and between the high-score group in vitamin C. Finally, in inorganic matter, significant differences were observed between the high-score group and the low-score group in phosphorus (*p* < 0.012), between the high- and low-score groups in potassium (*p* < 0.021), and between the high- and low-score groups in magnesium (*p* < 0.005).

## 4. Discussion

In sports, nutrition strategies have a great impact on improving performance. Therefore, it is important for athletes to judge their energy use and hydration according to their sports and characteristics and to develop nutrition plans and strategies considering them [10]. Nutrition considerations and requirements for individuals who are athletes with a disability may differ substantially from able-bodied athletes [12]; however, studies on specific nutritional requirements for optimal sports performance in athletes with disabilities are currently lacking.

As the Ministry of Health and Welfare declared the National Nutrition Control Act of 2010, the Ministry of Health and Welfare commissioned the Korea Nutrition Society to enact the Nutrition Standards and announced the first Nutrition Standards in 2015 and revised the second Korean Nutrition Standards in 2020 [13].

The recommended nutrient intake distribution among Koreans is 55–60% carbohydrates, 12–15% protein, and <15–30% fat that is based on linoleic acid, alpha-linolenic acid, and EPA + DHA [13]. Since the intake distribution of fat was not subdivided as recommended by Koreans in this study, it was excluded from the comparative analysis of %RDA of Koreans. However, in the total intake distribution, the fat intake ratio was within the recommended standard at 25.29% on average.

Athletes with a disability display variations in body composition, mobility, bone health, metabolic and neurological functions, all of which can significantly impact the athlete’s energy requirements [14]. Moreover, athletes with a disability are reported to have limited physical activity and lower total energy expenditure (TEE) than able-bodied athletes, resulting in less food consumption to maintain the energy balance [15,16]. However, negative energy consumption by athletes can lead to a lack of nutritional balance, which can increase metabolic processes, muscle function, and risk of injury [17]; therefore, awareness of nutritional intake is very important. To date, no guidelines for nutritional intake in individuals with spinal cord injury and athletes with a disability are available [18].

Macronutrients are divided into carbohydrates, fats, proteins, dietary fiber, and water. Carbohydrates play an important role in the storage and regeneration of glycogen in athletes [3]. Wheelchair athletes use many upper body muscles regardless of the cause of disability and use almost the same amount of muscle glycogen as able athletes, although the storage of muscle glycogen is significantly lower. Therefore, it is recommended to increase the proportion of carbohydrates before exercise [19]. Ruettimann et al. [20] reported the daily carbohydrate intakes of athletes with a spinal cord injury that range between 2.4 and 7.1 g/kg/d. Farkas et al. [18] reported that the total daily carbohydrate intake averaged 238 g/day and exceeded the minimum recommended value (130 g/day). Hence, greater energy intakes relative to energy requirements were highlighted regarding the general spinal cord injury population, and attention should be paid to multiple nutritional deficiencies resulting from large amounts of carbohydrate intake [18]. Dietary proteins affect muscle protein synthesis in the recovery process, and if not met, this can result in muscle loss and negative nitrogen balance [21]. Therefore, given the lack of recommendations for athletes with disabilities, the use and need for protein after exercise are similar to those of able athletes [22,23]. In contrast, a previous study suggested that compared to able athletes, athletes with disabilities will have lower protein intake because their muscle activity is lower and their overall energy requirements are reduced [24]. Thomas et al. [25] reported that the recommended daily allowance (RDA) of the able-bodied population is 0.8 g/kg body mass per day, and in able-bodied athletes, the amount of protein required each day ranges from 1.2 to 2.0 g/kg body mass. Considering that athletes with disabilities participating in this study have higher fat percentages and muscle mass than ordinary people, they need to consume higher amounts of carbohydrates and protein than the Korean recommended guideline, and the results of the study also confirmed higher carbohydrate and protein intakes than the RDA. In particular, the results of a comparative analysis of RDA of athletes with high energy demand and muscle protein synthesis cannot be generalized for carbohydrate and protein intakes. However, considering the characteristics of Koreans in this study, a comparison with the RDA can be a necessary step at the initial point of nutritional intake analysis of athletes with disabilities. It has also been reported that the level of intake and knowledge of water intake frequency and dietary fiber in athletes with disabilities is low [26]. The energy requirements of the disabled Korean athletes in this study averaged 70.6% of the recommended amount in Korea, showing sufficient intake of carbohydrates and proteins and less than the recommended amount of dietary fiber and moisture.

Vitamins and minerals act as important regulators of metabolic pathways. Some deficient vitamins and minerals result in poor athletic performance; problems have been identified in the catabolism of athletes, and increased excretion promotes excessive loss of trace nutrients [27]. Studies examining the effect of vitamin D supplementation on muscle function in athletes have reported inconsistent results. Farrokhyar et al. [28] found that hand grip strength increased significantly after 12 weeks of vitamin D supplementation. Zhang et al. [29], in a meta-analysis, examined the effect of vitamin D supplementation on muscle strength in athletes and reported that vitamin D supplementation positively affected lower limb muscle strength, but not upper limb muscle strength or muscle power, in athletes. However, intake of recommended amounts is important for athletes because the lack of vitamin D significantly affects muscle weakness; the frequency and duration of pain, injury, and disease; and, ultimately, results in poor motor performance [30]. In particular, frequent deficiencies can be seen in athletes with disabilities, with up to 51% of Canadian and American athletes reporting insufficient levels of vitamin D [31]. This deficiency may also affect the performance of exercise [22], especially in athletes with spinal cord injuries, which may increase the risk of osteoporosis because of excessive bone absorption and reduced bone formation. Vitamin E is also present in the cell membrane and plays a special role in preventing skeletal muscle damage as a major antioxidant [32]. Green-yellow vegetables, such as lettuce, spinach, and cabbage, the most common vegetable that Koreans eat, are high in vitamin K, and the high intake of vitamins E and K was also confirmed in this study. Vitamin K is known for its role in activating osteoblasts and promoting bone formation; therefore, it can be expected to have a preventive effect with respect to bone function and improvement of bone mass in osteoporosis [33].

Vitamin C, a water-soluble vitamin, can affect exercise performance because of muscle weakness, anemia, and delayed wound healing, and athletes are prone to excretion in large amounts of sweat and urine during intensive training [27]. In addition, vitamin B1 (thiamin) is a key nutrient in the carbohydrate and protein metabolism, and thiamine intake is observed in athletes and increases in high-intensity sports that require more energy [34]. In contrast, low thiamine intake has been reported in women’s gymnastics and wrestling, which are weight control sports that require low-calorie intake [27].

The high sodium intake levels of athletes in this study are thought to be partly attributed to fast-food-oriented frequent late-night snacks. Prior studies have shown that increased sodium intake has a negative effect on calcium retention in the body and results in sodium excretion in urine [35]. Iron is a major element needed to deliver oxygen to tissues and to use oxygen at the cellular and subcellular levels [36]. In particular, considering the exhaustion of sweat, it can be very important for players to eat iron-containing food. A previous study showed that 15–35% of female athletes are the most common to experience iron deficiency, although 5–11% of male athletes are also experiencing this deficiency [37]. Prior studies of wheelchair athletes have shown insufficient consumption of nutrients such as iron, phosphorus, and selenium, as well as niacin, riboflavin, thiamine, and vitamins A, E, B6, and B12, which suggests that nutrient supplementation should be recommended if a regular diet is insufficient [15]. Conversely, this study showed an average intake of more than the %RDA in vitamins E, B6, and B12, riboflavin, phosphorus, iron, iodine, and selenium, which are likely to be deficient in athletes with disabilities.

In general, indicators evaluating the quality of meals are used to assess how well the dietary recommendations or guidelines are being followed and developed to evaluate permanent, food-/food-group-oriented, or mixed nutrient and food group changes [38]. Among them, the nutrition quotient used in this study is a simple, proven checklist that comprehensively evaluates the nutritional status of individuals, groups, and the quality of meals [39]. Recently, the Korean Nutrition Society has developed a nutrition index for each stage of life, including pre-school children (NQ-P) [40], adolescents (NQ-A) [41], and the elderly (NQ-E) [42]. The nutrition quotient for outcomes used in this study (NQ-A) is appropriately used to compare the nutritional conditions of manufacturing workers [43] and the effects of self-management of university athletes [44].

In this study, the scores corresponding to the high- and middle-score groups by NQ-A grade were higher than those of adults nationwide [9]. The NQ-A survey of adults across the country showed a balanced score of 38.6, a variety of 55.9, ablation of 67.1, and dietary behavior of 47.0, with a similar score as the high-score group in this study. Therefore, it can be confirmed that the nutrition management level and awareness of the national athletes with disabilities participating in this study is high. A comparative analysis of the NQ-A detailed factor scores showed significant differences between NQ-A grades in variety, moderation, and diet except for balance, low-score group levels of fruit, dairy, nuts, fish intake, variety indicating vegetables, water intake frequency, picky levels, nutrition indications, health, dietary efforts, and nutrition.

Results of %RDA comparison analysis according to NQ-A score grades showed significant differences between the higher-score group for dietary fiber, vitamin E, vitamin C, and thiamine among the macronutrients and phosphorus, potassium, and magnesium among the minerals. Dietary fibers play a role in bowel function, promote bowel activity, reduce cholesterol absorption [40], and inhibit blood sugar absorption, thus maintaining increased postprandial blood sugar levels and long-term satiety in the small intestine [45]. A previous study suggested that dietary fiber levels need to be considered in groups other than the high-score group of NQ-A, such as insufficient dietary intake in athletes with disabilities [19].

In this study, the vitamin E intake rate was higher than the %RDA. In contrast, the vitamin C intake rate showed a lower overall average than the %RDA. Vitamin C affects continuous exercise associated with fatigue [27], and considering the nature of water-soluble vitamins that are not stored in the body, it is necessary to recommend a sufficient intake. Thiamine and phosphorus also showed higher intake rates compared to the %RDA, and a significant reduction was observed in the low-score group of NQ-A. Therefore, since phosphorus is an important nutrient contributing to soft tissue or cell membrane components and energy metabolism, acid–base balance of body fluids, and bio-signaling [46], it may be important to recommend a sufficient intake to phosphorus-deficient athletes.

Finally, significant differences between potassium and magnesium levels were identified between the NQ-A score groups. Besides the potassium intake of the high-score group, the recommended amount is insufficient. Magnesium is a component of bone and teeth in the body and is an important factor affecting vitamin D metabolism [47], while phosphate is known to form magnesium and insoluble salts that inhibit the absorption of magnesium [48]. Therefore, nutritional awareness and recommendations for an inorganic balance are needed.

Since there are still no nutritional guidelines for disabled athletes, the RDA of non-athletes was used as the baseline in this study, but caution may be needed when applying these results directly to disabled athletes. Therefore, to improve the health and performance of athletes with disabilities, a summary of intakes and a study of nutritional guidelines are needed, considering the body composition, sports performance, exercise intensity, and total energy expansion.

## 5. Conclusions

This study evaluated the nutrition intake and nutrition index of 21 athletes with disabilities preparing for the Tokyo Paralympics. As a result of this study, carbohydrate and protein intake was higher than %RDA, and intake of vitamin E, thiamine, riboflavin, vitamin B6, and vitamin B12 was also higher. As for the NQ-A, 10 athletes belonged to the high-score group, followed by 6 athletes in the middle-score group and 5 athletes in the low-score group. In particular, as the results of comparing dietary intake between the three groups showed, the low NQ-A score group had significantly lower intake compared to the %RDA for vitamin E (67.1%), vitamin C (26.3%), and potassium (42.8%). However, with most nutrients, nutritional intake deficiency was not shown to be a problem.

Based on the results of this study, we would like to further our research in order to provide basic data that can be used as nutrition guidelines by performing nutritional research and analysis, including personal characteristics and events of athletes with disabilities.

## Figures and Tables

**Table 1:** General characteristics of Korean athletes with physical disabilities.

	*n* = 21	
Gender	Males	16
	Females	5
Age	19–29	3
	30–49	12
	50–64	6
Sport	Shooting	3
	Archery	4
	Boccia	3
	Table tennis	2
	Athletics	2
	Swimming	1
	Judo	2
	Track cycling	2
	Badminton	3
National Team career	Less than 5 years	6
	More than 5 years	15
Type of disability	Visual impairments	2
	Spinal cord disorder	16
	Cerebral palsy	3
Total energy intake ratio	Carbohydrate	58.97%
	Protein	15.74%
	Fat	25.29%

**Table 2:** Comparison of average dietary intake and %RDA (KDRIs, 2020) of Korean nationals with disabilities.

Variables			Males (*n* = 16)			Females (*n* = 5)			Total Mean Intakes	Total Mean %RDA
			Mean Intakes	RDA	%RDA, %AI	Mean Intakes	RDA	%RDA, %AI		
**Macronutrient intakes**										
Total calories (kcal)		19–29	1448.75	2600	56%	-	-	-	1622.59	70.9%
		30–49	1806.86	2500	72%	1346.25	1900	74%		
		50–64	1833.54	2200	83%	1155.51	1700	68%		
Carbohydrate (g)			233.48	130	180%	164.32	130	126%	217.01	166.9%
Protein (g)		19–49	70.04	65	108%	56.45	50	113%	67.94	112.3%
		50–64	72.97	60	122%	61.52	50	123%		
Dietary fiber (g)			17.67	30	59%	13.65	20	68%	16.71	61.1%
Water (g)		19–29	423.85	1400	30%	-	-	-	725.84	60.6%
		30–49	863.16	1300	66%	732.02	1000	73%		
		50–64	862.75	1200	72%	595.82	900	66%		
**Vitamins**										
Fat-soluble vitamins	A (µg)	19–49	409.03	800	51%	324.80	650	50%	424.91	56.5%
		50–64	702.18	750	94%	283.24	600	47%		
	D (µg)		3.81	10	38%	2.56	10	26%	3.51	35.1%
	E (mg)		14.57	12	121%	9.83	12	82%	13.44	112.0%
	K (µg)		72.50	75	97%	41.52	65	64%	65.12	88.8%
Water-soluble vitamins	C (mg)		81.14	100	81%	38.48	100	38%	70.98	70.9%
	Thiamine (mg)		1.63	1.2	136%	0.99	1.1	90%	1.47	124.6%
	Riboflavin (mg)		1.54	1.5	103%	1.10	1.2	92%	1.43	100.2%
	Niacin (mg)		12.98	16	81%	7.59	14	54%	11.69	74.8%
	B6 (mg)		1.76	1.5	118%	1.25	1.4	89%	1.63	110.6%
	Folate (folic acid) (µg)		413.65	400	103%	289.77	400	72%	384.15	96.0%
	B12 (µg)		2.94	2.4	122%	2.77	2.4	116%	3.51	120.7%
	Pantothenic acid (mg)		4.20	5	84%	3.22	5	64%	3.95	79.2%
	Biotin (µg)		1.59	30	5%	1.17	30	4%	1.48	4.9%
**Minerals**										
Macrominerals	Calcium (mg)	19–49	367.13	800	46%	608.16	700	87%	432.25	55.74%
		50–64	702.48	750	94%	393.62	800	49%		
	Phosphorus (mg)		1019.19	700	146%	701.56	700	100%	829.27	118.3%
	Sodium (mg)		3439.03	1500	229%	2643.89	1500	176%	3249.71	216.6%
	Chlorine (mg)		90.78	2300	4%	320.08	2300	14%	145.37	6.3%
	Potassium (mg)	19–49	2321.87	3500	66%	1776.74	3500	51%	2297.16	71.3%
		50–64	3057.48	2400	127%					
	Magnesium (mg)	19–29	42.40	360	12%	44.47	280	16%	78.08	22.0%
		30–64	99.25	370	27%					
Trace Minerals	Iron (mg)	19–49	14.00	10	140%	7.79	14	56%	14.17	146.5%
		50–64				19.37	8	242%		
	Zinc (mg)		9.34	10	93%	6.23	8	78%	8.59	89.6%
	Copper (mg)		563.04	850	66%	484.58	650	75%	544.36	68.3%
	Fluorine (mg)	19–49	0.06	3.4	2%	0.01	2.7	0%	0.04	1.2%
		50–64	0.01	3.2	0%	0.01	2.6	0%		
	Manganese (µg)		1.58	4	39%	1.45	3.5	41%	1.54	39.9%
	Iodine (µg)		238.65	150	159%	138.46	150	92%	214.79	143.2%
	Selenium (µg)		72.61	60	121%	55.57	60	93%	68.54	114.2%
	Molybdenum (µg)	19–49	2.23	30	7%	3.00	25	12%	2.13	7.8%
		50–64				1.07	14	8%		

KDRIs, Dietary Reference Intakes for Koreans (2020; http://www.kns.or.kr/FileRoom/FileRoom_view.asp?idx=108&BoardID=Kdr; accessed on 8 April 2021); RDA, recommended daily allowance; AI, adequate intake; %RDA, the percentage of the RDA value is expressed as percentage of RDA; %RDA = nutrition intake/RDA(sex, age) × 100.

**Table 3:** Nutrition quotient for adults (NQ-A) and scores according to detailed factors.

Variables	High-Score Group (*n* = 10) (a)	Middle-Score Group (*n* = 6) (b)	Low-Score Group (*n* = 5) (c)	F	*p*	*Scheffe*
Overall NQ-A	66.73 ± 3.40	51.18 ± 3.48	42.22 ± 1.07	118.399	0.001 **	c < b < a
NQ-A components						
Balance	44.95 ± 15.58	34.91 ± 14.85	25.62 ± 12.66	2.977	0.076	
Diversity	66.24 ± 9.35	53.45 ± 10.83	28.82 ± 11.34	22.228	0.001 **	c < ab
Moderation	82.26 ± 9.35	66.01 ± 11.70	72.72 ± 4.31	5.415	0.014 *	b < a
Dietary behavior	71.46 ± 5.97	46.46 ± 10.83	34.06 ± 8.73	39.573	0.001 **	bc > a

* *p* < 0.05, ** *p* < 0.001. Scheffe’s post hoc test (a) high-score group; (b) middle-score group; (c) low-score group.

**Table 4:** Comparative analysis of %RDA (KDRIs, 2020) according to nutrition quotient for adults (NQ-A) score groups of national athletes with disabilities.

Variables		Quotient-Adults (NQ-A)					
		High-Score Group (*n* = 10) (a)	Middle-Score Group (*n* = 6) (b)	Low-Score Group (*n* = 5) (c)	F	*p*	*Scheffe*
**Macronutrient intakes (%RDA)**							
Total calories		79.97 ± 11.85	62.33 ± 13.49	63.16 ± 13.15	4.908	0.020 *	b < a
Carbohydrate		187.30 ± 54.37	155.67 ± 27.33	139.79 ± 29.99	2.279	0.131	
Protein		126.75 ± 45.57	101.00 ± 21.28	97.08 ± 19.32	1.608	0.228	
Dietary fiber		78.92 ± 32.08	44.33 ± 12.06	45.72 ± 15.99	4.941	0.019 *	b < a
Water		67.00 ± 13.26	56.00 ± 19.62	53.68 ± 21.50	1.302	0.296	
**Vitamins (%RDA, %AI)**							
Fat-soluble vitamins	A	74.64 ± 54.78	41.67 ± 22.13	38.30 ± 18.54	1.829	0.189	
	D	42.30 ± 28.33	35.00 ± 17.45	21.17 ± 17.24	1.348	0.285	
	E	142.35 ± 56.19	99.00 ± 25.63	67.07 ± 26.14	5.311	0.015 *	c < a
	K	107.37 ± 77.47	84.33 ± 61.38	57.22 ± 42.35	0.962	0.401	
Water-soluble vitamins	C	114.68 ± 76.64	35.33 ± 13.66	26.28 ± 14.19	6.050	0.010 **	bc < a
	Thiamine	158.26 ± 57.69	103.83 ± 22.12	82.59 ± 27.77	5.768	0.012 *	c < a
	Riboflavin	110.73 ± 46.61	92.17 ± 34.23	89.07 ± 11.08	0.735	0.493	
	Niacin	83.98 ± 37.65	77.17 ± 17.95	53.72 ± 22.78	1.696	0.211	
	B6	118.16 ± 43.68	118.67 ± 60.16	86.01 ± 32.06	0.911	0.420	
	Folate (folic acid)	110.94 ± 54.85	97.67 ± 38.70	64.36 ± 24.30	1.768	0.199	
	B12	120.50 ± 35.53	128.00 ± 41.78	120.76 ± 35.75	0.234	0.794	
	Pantothenic acid	88.83 ± 23.79	75.33 ± 27.26	64.88 ± 8.96	2.012	0.163	
	Biotin	7.44 ± 10.09	2.17 ± 4.83	3.43 ± 2.13	1.027	0.378	
**Minerals (%RDA, %AI)**							
Macrominerals	Calcium	65.89 ± 27.74	44.66 ± 14.88	48.75 ± 26.88	1.656	0.219	
	Phosphorus	145.27 ± 40.91	108.66 ± 39.95	76.17 ± 21.79	6.027	0.010 **	c < a
	Sodium	259.86 ± 121.75	201.83 ± 44.27	147.86 ± 40.75	2.622	0.100	
	Chlorine	5.86 ± 6.18	2.17 ± 1.47	12.22 ± 25.05	0.880	0.432	
	Potassium	96.15 ± 42.60	53.83 ± 13.61	42.81 ± 9.95	6.152	0.009 **	c < a
	Magnesium	26.80 ± 8.72	22.50 ± 5.61	11.99 ± 4.64	7.107	0.005 **	c < a
Trace minerals	Iron	184.45 ± 116.95	124.17 ± 28.65	97.63 ± 64.71	1.834	0.188	
	Zinc	97.84 ± 25.67	93.17 ± 25.17	69.12 ± 9.01	2.724	0.093	
	Copper	78.42 ± 40.47	56.67 ± 11.71	62.13 ± 29.22	0.968	0.399	
	Fluorine	0.88 ± 2.18	2.50 ± 3.78	0.41 ± 0.43	1.113	0.350	
	Manganese	45.80 ± 39.39	33.17 ± 20.93	36.36 ± 35.50	0.290	0.752	
	Iodine	190.02 ± 205.89	89.17 ± 55.66	114.48 ± 202.98	0.698	0.511	
	Selenium	124.30 ± 58.32	104.53 ± 27.60	105.54 ± 29.22	0.457	0.640	
	Molybdenum	8.84 ± 5.63	6.83 ± 5.98	7.00 ± 4.84	0.316	0.733	

* *p* < 0.05, ** *p* < 0.01. Scheffe’s post hoc test (a) high-score group; (b) middle-score group; (c) low-score group. KDRIs, Dietary Reference Intakes for Koreans (2020; http://www.kns.or.kr/FileRoom/FileRoom_view.asp?idx=108&BoardID=Kdr; accessed on 8 April 2021); RDA, recommended daily allowance; AI, adequate intake; %RDA, the percentage of the RDA value is expressed as percentage of RDA; %RDA = nutrition intake/RDA(sex, age) × 100.
